# Evaluation of a Mobile App to Enhance Relational Awareness and Change During Cognitive Analytic Therapy: Mixed Methods Case Series

**DOI:** 10.2196/19888

**Published:** 2020-12-18

**Authors:** Stephen Kellett, Katherine Easton, Martin Cooper, Abigail Millings, Melanie Simmonds-Buckley, Glenys Parry

**Affiliations:** 1 Sheffield Health & Social Care NHS Foundation Trust University of Sheffield Sheffield United Kingdom; 2 University of Sheffield Sheffield United Kingdom; 3 Sheffield Hallam University Sheffield United Kingdom; 4 Catalyse Sheffield United Kingdom

**Keywords:** cognitive analytic therapy, case series, effectiveness, outcome, eHealth, app, awareness, mHealth, innovation, therapy

## Abstract

**Background:**

There has been a lack of technological innovation regarding improving the delivery of integrative psychotherapies. This project sought to evaluate an app designed to replace previous paper-based methods supporting relational awareness and change during cognitive analytic therapy (CAT).

**Objective:**

We aimed to assess patients’ and therapists’ experience of using the technology (ie, the “CAT-App”) and to evaluate the relationship between app usage and clinical outcome.

**Methods:**

The design was a mixed methods case series. Patients completed the Clinical Outcomes in Routine Evaluation-Outcome Measure pre- and post-CAT. Mood data plus the frequency and effectiveness of relational awareness and change were collected via the app. Therapists and patients were interviewed about their experiences using the app.

**Results:**

Ten patients (treated by 3 therapists) were enrolled; seven completed treatment and 4 had a reliable improvement in their mental health. App usage and mood change did not differ according to clinical outcome, but there was a statistically significant difference in app usage between completers and dropouts. The qualitative themes described by the therapists were (1) the challenge of incorporating the technology into their clinical practice and (2) the barriers and benefits of the technology. Clients’ themes were (1) data protection, (2) motivation and engagement, and (3) restrictions versus flexibility.

**Conclusions:**

The CAT-App is capable of supporting relational awareness and change and is an upgrade on older, paper-based formats. Further clinical evaluation is required.

## Introduction

Mental health disorders are the single largest cause of health-related economic burden worldwide [1], and, globally, there is mounting pressure on health care providers to ensure rapid access to effective, evidence-based, organizationally efficient, and cost-effective psychological interventions. Digital technology bridges the demand-supply gap by offering easily accessible, flexible, and personalized support [2], thereby creating the possibility to affect large-scale and low-cost change in public mental health [3]. The World Health Authority has therefore championed development and evaluation of electronic mental health care [4]. Electronic mental health services are defined by the provision of digital interventions via mobile apps/tablets and online, web-based programs [5], with interventions either being delivered as standalone technologies or being integrated into face-to-face therapy [6]. The technology allows symptom monitoring, provides psychoeducation, and promotes ongoing self-management strategies [7]. The technology also collects in situ assessments of mental health symptoms, which are an ecologically valid source of naturalistic research data [8]. Online, web-based programs have generated a large evidence base in support of their efficacy and effectiveness [9], while the evidence in support of mental health apps is still under development [10].

Evident enthusiasm for electronic mental health innovation is somewhat tempered by evidence that certain mental health conditions (eg, depression, paranoia, or psychosis) create problems with engagement and may weaken users’ trust in the technology itself [11]. “Technology push” also occurs when the commercial concerns of digital health care companies trump the wants/needs of patients and also challenges the values of clinicians [2]. The lack of sufficient depth of clinical and academic collaboration regarding electronic mental health innovation has been highlighted as a key feature of “technology push” [12]. The speed at which electronic mental health can be developed also threatens to ignore (or be ignorant of) robust methods for treatment development and associated evaluation [13]. The content of some electronic mental health apps has also been criticized for not being grounded in sufficient theory [14] and for the fact that the outcomes achieved during clinical trials are rarely replicated in routine service delivery settings [15]. Although a plethora of electronic mental health apps are readily available for the treatment of a variety of disorders and in a variety of contexts, questions can linger concerning a lack of evidence regarding feasibility, safety, clinical effectiveness, and efficacy [12]. The potential for technological innovations to outstrip the co-development of a sufficiently robust evidence base therefore risks a loss of confidence/trust from both clinicians and patients [16].

The electronic mental health field is currently also dominated by apps reflecting the changing methods of cognitive behavioral therapy (CBT), in part because of the ease with which those changing methods can be translated into app content [12]. For example, a recent review of CBT-based apps for the management of depression found that 31 apps were available [3]. However, the acceptability of the CBT approach is not universal and so uptake and dropout rates can be very variable [17]. There are no known examples of apps that support the work of integrative and psychodynamic psychotherapies. A widely practiced integrative psychotherapy is cognitive analytic therapy (CAT) [18]. CAT is a time-limited and relationally-driven psychotherapy [18,19], which has a generally high quality evidence base [20]. A recent meta-analysis showed that CAT had moderate-to-large effects on global functioning, interpersonal difficulties, and depression in practice-based studies, while during clinical trials the pooled effect size showed a small but significant treatment effect in favor of CAT over controls [21].

CAT is an integration of personal construct and object relations theory [22]. Internalized early object relations are termed “reciprocal roles” which influence/limit relational repertoires [23]. “Target problems” are the diagnosis/presenting problem reframed in relational themes, and “target problem procedures” describe the present day “traps” (ie, vicious circles), “snags” (ie, self-sabotage), and “dilemmas” (ie, “either/or” dilemmas) maintaining relational problems [18]. The procedural sequence object relations model anchors target problem procedures in reciprocal role activation [19]. CAT has been recently summarized into a competency framework [24]. CAT uses a 3-phase approach: (1) an initial “reformulation” stage during which target problems and target problem procedures are summarized in narrative and diagrammatic reformulations, (2) a middle “recognition” stage facilitating self and relational awareness, and (3) a final “revision” stage that is focused on change. Therapeutic change during CAT is founded on effective relational awareness [25].

The CAT-App had the aim of digitizing the in-session and between-session recognition and revision tasks that constitute the middle and final phases of the therapy [26]. Previously, within each session, the CAT therapist and patient reviewed and rated recognition and revision of target problems and target problem procedures on paper-based rating sheets. Patients were also previously provided with a variety of paper-based target problem procedure recognition tools for use between-sessions. These between-session tools have been criticized for potentially creating stigma or embarrassment due to the difficulty of completing the tools surreptitiously [27]. The CAT-App was a proposed improvement on between-session methods, as the technology provides an unobtrusive method of collecting ideographic data in real time, contains dynamic feedback on recognition and revision trajectories, contains narrative and diagrammatic reformulations, and also captures users’ current moods. The potential in-session time saving would be that CAT therapists would be able to open the app during sessions and review ongoing recognition and revision rather than making single ratings within the session. The overall aims of this study were to evaluate the relationship between app usage and clinical outcome and also to attain feedback from patients and therapists on their experiences of the technology.

## Methods

### Design, Service, Therapists, and Ethics

The design was a mixed methods case series evaluation, and ethical approval for the study was awarded from the University of Sheffield Ethics Committee (ethics reference number: 012217). The study setting was a private sector CAT clinic based in the United Kingdom. This is a not-for-profit service that offers CAT to people that cannot or choose not to access CAT through their local National Health Service mental health services. The service is staffed by therapists that are either accredited CAT practitioners or CAT psychotherapists. All the therapists (N=3) were in monthly clinical supervision for their CAT work. All were experienced therapists as each had completed more than 11 post-qualification years of CAT practice. Two were female CAT practitioners and one was a male CAT psychotherapist. Therapists underwent a brief (2 hour) training session on how to use the technology and were given a user manual.

### Inclusion and Exclusion Criteria

Inclusion criteria were (1) willingness to engage in CAT, (2) owning a smart phone, and (3) willingness to use the app. Eligibility was not dependent on receiving therapy. Participants were not excluded if they had previously received other forms of psychotherapy. Participants were not required to have any previous experience of using health technology. The exclusion criteria were (1) currently misusing substances to a significant degree, (2) currently frequently self-harming, (3) high on-going risk of suicide, and (4) currently posing a risk to another person to a significant degree.

### Outcome Measure

The Clinical Outcomes in Routine Evaluation-Outcome Measure (CORE-OM) was used to evaluate clinical outcome, as this is a valid and reliable measure of psychological distress commonly used in psychotherapy outpatient clinics [28]. The measure has good concurrent [28] and discriminant validity [29], sound internal and test-retest reliabilities [28], and is sensitive to psychotherapeutic change [29]. The CORE-OM was completed at assessment and termination of CAT. Outcomes on the CORE-OM were evaluated regarding the degree and clinical significance of change. The degree of change was assessed with the reliable change index [30]. The reliable change index tests for the degree of change required to be considered reliable, rather than that expected to occur by chance. The pre-post total CORE-OM score needs to change by 5 or more to assign a reliable improvement outcome (or reliable deterioration if the pre-post score increases by 5 or more). Clinically significant change [30] occurs when the pre-post outcome shifts in classification from “caseness” to “non-caseness.” The clinical cut-off score on the CORE-OM is 10. Simultaneous reliable and clinically significant change is a credible index of “recovery” in routine practice [31]. The pre-post effect size for the case series was computed and interpreted using Cohen’s power primer, where d≥0.20 is a “small” effect, d≥0.50 is a “medium” effect, and d≥0.80 is a “large” effect [32].

### Qualitative Interviews

Semistructured interviews (45-60 minutes) were conducted with patients and with therapists. Interviews took place within 1 month of treatment being completed. Interviews were recorded using an encrypted digital audio recorder and transcribed verbatim. Interviews were conducted at a site convenient to the therapist or patient. Patient interviews explored the experiences of using the app, perceived benefits and barriers to usage, acceptability, perceived impact on recognition and revision of target problems and target problem procedures, and tolerability/burden of the measures embedded in the app. Therapist interviews explored experiences of incorporating the technology within the care pathway, perceived benefits and barriers to use of the CAT-App, acceptability of the technology, perceived impact on recognition and revisions efforts, and burden/tolerability of the measures. Data were analyzed using thematic analysis [33].

### Treatment

CAT is a time-limited psychotherapy delivered in 8, 16, or 24 session contracts according to diagnosis and severity [18]. Sessions were weekly and lasted for 50-60 minutes. The CAT delivered contained the 3 stages consistent with the clinical model: (1) reformulation (2), recognition, and (3) revision. Reformulation consisted of an assessment phase enabling a narrative reformulation naming target problems and their developmental origins, how the problem is maintained (ie, target problem procedures), hypotheses about the manner in which the participant might experience the help offered by the therapist, and finally acknowledging any issues concerning the ending of the therapy. Recognition was marked by methods to enhance self-awareness of problematic states/roles/procedures, via production of a sequential diagrammatic reformulation and associated relational awareness monitoring as between-session homework. Narrative and diagrammatic reformulations were made accessible to participants on the CAT-App. As such, participants had immediate access to their personal reformulations to support relational recognition efforts (which was not possible in the previous paper-based approach). Revision focused on application of change methods (“exits,” in the language of CAT) which were bespoke to the participant, their individual reformulation, and their zone of proximal development [34]. In keeping with CAT practice, changes were visually labelled as exits on sequential diagrammatic reformulations [18] and so further change-based sequential diagrammatic reformulations that also had exits added were uploaded to the app. In the final session, both patient and therapist produced and shared “goodbye letters” [18]. The function of these letters is to reflect on the ending of the therapy and what this means to the patient, name the dominant relational patterns that occurred within the therapeutic relationship, name abandonment feelings, mark progress, and identify relapse prevention strategies [19].

### The CAT-App: Description and Usage Data

The co-design process of the CAT-App has been previously described [35]. Participants were not advised nor prompted to use the app in terms of a specified frequency but, in keeping with the model, were encouraged simply to notice and record problematic roles and procedures. The app had the ability to store and display the individual narrative reformulation, sequential diagrammatic reformulation, and goodbye letters. Patients opened the app to rate the degree to which they were recognizing (0-100 scale from ineffective recognition to effective recognition) their individual target problems and target problem procedures. If the patient was in the latter revision stages of CAT, then they would also rate the effectiveness of the degree to which they have revised (ie, changed) the associated target problem and target problem procedure. Feedback on current and all previous attempts at recognition and revision were graphed in order to visibly provide and display feedback. Patients were also allowed to write electronic notes on their recognition and revision efforts. When patients rated their current mood on 0-10 slider scales, a graph plotted current rating against previous ratings.

The number of times that the participants opened the app and the clinical details of the target problems and target problem procedures were recorded. Current mood state (rated 0-10) was collected on the following dimensions: sad-happy, anxious-calm, and excited-bored (reverse scored). These items were chosen to measure the pleasure (valence) and arousal (activation) dimensions of core affect [36], specifically, pleasure (sad-happy), pleasant deactivation (anxious-calm), and pleasant activation (bored-excited). Mood scores were grouped into observation quartiles to track mood changes over the course of treatment. Mean mood scores, variability of mood scores, and app usage were compared for patients that met criteria for reliable change versus nonreliable change.

## Results

The results are presented in two parts to meet the objectives of the study. The first section presents the quantitative results in terms of describing the sample, dropout rate, target problem and target problem procedure recognition and revision ratings, the clinical outcomes, and relationship between app usage and both clinical outcome and mood change over treatment time. The second section concerns the qualitative results of therapists’ and patients’ experience of CAT-App usage.

Ten patients initially consented to the study and 3 (30%) dropped out early during treatment (<3 sessions). Therefore, the case series (ie, the computer sample) consisted of 7 patients, 4 male and 3 female. The mean age of the patient sample was 34.71 years (SD 7.18). The presenting problems were mixed anxiety and depression (n=5), borderline personality disorder (n=1), and narcissistic personality disorder (n=1). Treatment duration was 16 sessions for the mixed anxiety and depression cases and 24 sessions for the personality disorder cases and is consistent with session allocation within the CAT model [18]. Six of the 7 completers were “cases” at assessment on the CORE-OM. Table 1 describes the 21 target problems rated in the app. In terms of the associated target problem procedures, 12 were traps, 6 were dilemmas, and 2 were snags. These were described in their original individual long form on the CAT-App.

**Table 1:** The target problems of the participants and associated theoretical concepts.

Study participant ID	Target problem 1	Target problem procedure	Target problem 2	Target problem procedure	Target problem 3	Target problem procedure
1	Poor pacing	Dilemma	Grandiosity	Dilemma	Excessive drinking	Trap
2	Self-critical	Trap	Poor pacing	Dilemma	People pleasing	Snag
3	Seeing self as weak	Trap	Anxiety	Dilemma	Self-critical	Trap
4	Poor self-care	Trap	Anxiety	Trap	Emotionally cutoff	Dilemma
5	Isolating self	Snag	Social anxiety	Trap	Procrastination	Trap
6	Performance anxiety	Trap	Anxiety	Trap	Feeling judged by others	Trap
7	Boom or bust	Dilemma	Untrusting of people	Snag	Emotional suppression	Trap

For example, for the patient with narcissistic personality disorder, the target problem was “how I feel about myself keeps flipping between bigging myself up or feeling like a loser” with the associated dilemma “Either I feel superior to other people and am a bit contemptuous of them or I feel they are looking down on me and I think I'm rubbish”. Please note that the exact wording of these examples have been altered to protect patient anonymity, but without altering the clinical meaning

**Figure 1:**
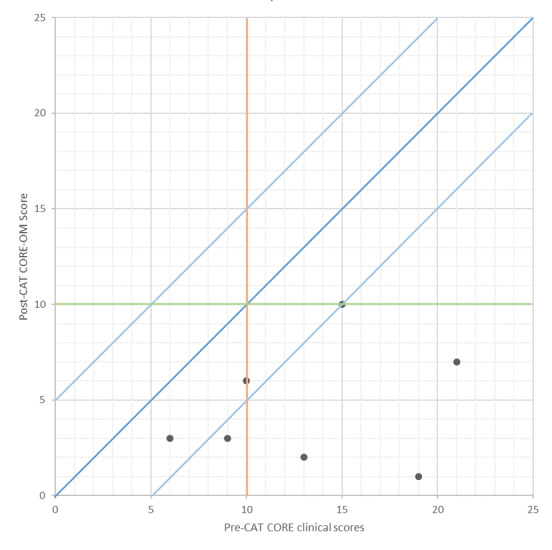
Jacobson plot of clinical outcomes. CAT: cognitive analytic therapy. CORE-OM: Clinical Outcomes in Routine Evaluation-Outcome Measure.

In terms of usage data, the completer sample used the technology on average 119.86 times (SD 97.98) ranging from 11-239 occurrences. The dropout sample used the technology on average 5.00 times (SD 2.71) ranging from 3-9 occurrences. There was a significant difference in usage between completers and dropouts (t=2.29, *P*=.04). Figure 1 describes a CORE-OM Jacobson plot of the clinical outcomes for the 7 completers. There was a significant reduction in psychological distress between start (M=13.02, SD 5.41) and completion (M=4.68, SD 3.19) of treatment (z=4.50, *P*=.01), with a large effect size (d+=1.51). One of the patients remained a “case” on the CORE-OM at termination; 4 had a reliable reduction in distress (3 of whom also were in the community norm on the CORE-OM by end of treatment). The “recovery rate” was therefore 3/7 (42.85%).

Table 2 compares the percentage of ineffective and effective recognition and revision ratings for the clinical outcome groups. The completer sample is split between those who had no reliable change on the CORE-OM (n=3; 246 app observations, range 11-235) versus those who had a reliable pre-post reduction on the CORE-OM (n=4; 572 app observations, range 51-239). Usage (number of observations) did not significantly differ between patients who experienced reliable change (M=143, SD 82.82) and those that did not (M=89; SD 126.54; *t*(5)=0.689, *P*=.521). Table 3 presents the mean mood scores for the completer sample and then those patients with reliable change and those that did not change.

**Table 2:** Recognition by clinical outcome (N=7).

Target Problem	No change (n=3 patients and 246 app observations)				Reliable change (n=4 patients and 572 app observations)			
	Ineffective recognition rating, %	Effective recognition rating, %	Ineffective revision rating, %	Effective revision rating, %	Ineffective recognition rating, %	Effective recognition rating, %	Ineffective revision rating, %	Effective revision rating, %
								
1	59.3	40.7	82.6	17.4	45.6	54.4	61.1	38.9
2	50.6	41.6	79.7	20.3	50.4	49.6	47.9	52.1
3	40.8	59.2	72.5	27.5	38.7	61.3	60.4	39.6

**Table 3:** Mean mood scores (SD) by clinical outcome group.

Current mood state	Overall value (N=7) (SD)	Reliable change value (n=4) (SD)	Nonreliable change value (n=3) (SD)	*t*-score (*P* value)
Anxious-calm	3.44 (1.06)	3.11 (0.91)	4.10 (1.36)	1.099 (.334)
Bored-excited	6.28 (0.67)	6.47 (0.65)	5.90 (0.72)	0.993 (.377)
Sad-happy	3.65 (0.92)	3.41 (0.82)	4.15 (1.20)	0.928 (.406)

**Figure 2:**
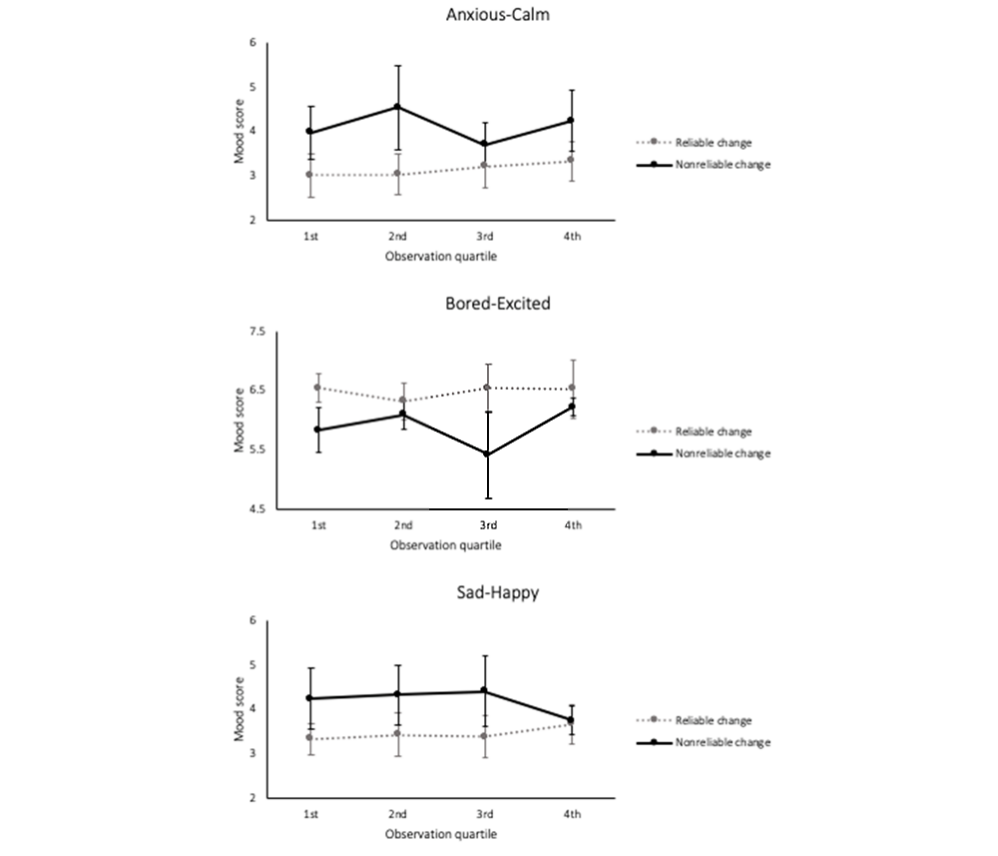
CAT-app mood scores grouped by observation quartiles (error bars=SD).

Mood scores did not significantly differ according to clinical outcome. Figure 2 plots the observation quartile mood scores throughout treatment for the clinical outcome groups. Levene’s tests and associated t-tests for each phase failed to identify any significant differences in mood means and variances (SDs) between the clinical outcome groups (ie, *P*>.05 in all 4 comparisons of means and variances on each mood scale).

Of the 7 patients, 6 were interviewed (ie, a single patient was uncontactable to interview) and all 3 therapists were interviewed. Two main themes emerged from therapists: (1) incorporating the technology into clinical practice and (2) the perceived barriers and benefits to the technology use. Table 4 provides the themes, subthemes, and example quotes from the therapists and patients.

**Table 4:** Experience of the CAT^a^-App: qualitative themes.

User experience, themes, and subthemes			Evidence
**Therapists' experience**			
	**Incorporating the app into clinical practice**		
		Promise of the app	“Some clients write a lot, others forget the paper or leave papers at school or home, especially younger clients.” (ID1)
		Using the app	“I introduced the app in the first session and then we would wait until session 5 before using it because it takes time to define the target problem procedures. I just cut and pasted from the letter straight into the app.” (ID3)
		Take up by patients	“All the clients were digitally literate. There was no hand holding and I told them to get back to me if they had any issues.” (ID3)
	**Perceived threats and benefits**		
		Non-equivalence	“I didn’t want the app taking any decisions from therapists. I don’t want to be a virtual therapist.” (ID2)
		Anxiety regarding the app	“It felt like extra work [...] I was worried in case I uploaded the wrong information to the wrong account.” (ID2)
		Supporting self-reflection	“The app allows the client to become more of an observer of their behaviors than does the paper version.” (ID1)
		Anxiety regarding data protection	“We need to know exactly where the client’s data is stored and what happens to that data once the client is discharged, for example.” (ID2)
**Patients' experience**			
	**Data protection and storage**		
		Trust	“I trusted the therapist and the app” (ID4) “I’m more bothered about bank details than emotional stuff” (ID9)
		Normality of data sharing	“What’s the worst that could happen?” (ID4) “I’m happy with the Ts & Cs. I don’t really read them anyway” (ID5)
		Server felt safe	“It’s better than paper, I can’t lose it” (data) (ID6) “It’s good the data isn’t on my phone. It means I can only work online but at least nothing is saved on my phone” (ID 5)
	**Motivation and engagement**		
		Initial excitement	“I like the idea, I set it all up straight away” (ID4) “Initially I thought it was a great idea, better than using paper” (ID 7) “I was excited to try, I’ll give these things a go” (ID 8)
		Reminders	“I liked it but I’m not driven to use it, I needed more discipline. Maybe nudges as well as reminders would help” (ID4) “You can set reminders to use it but it would be good to have thought provoking messages as well, relating to CAT” (8)
		Habit forming	“I didn’t have access for 3 weeks when I was abroad. I got used to using paper and just stuck with that” (ID7) “It made me more conscious of my targets in the day. Knowing I could just not something down or reflect. I found it useful” (ID5)
	**Restrictions and flexibility**		
		Online/offline access	“It’s good that data is stored on a server but that means I can only use it if I’m online. It needs a save and sync option” (ID5)
		Convenience	“It was good to make notes as and when I needed to” (ID8) “It was a great place to deposit feeling and later revisit them in the evening to make sense of them. You forget sometimes in the middle of the day and then you don’t feel as extreme later. It was good to note things quickly to analyze” (ID4) “I liked how my therapist could access things. It was on way but she could look at things with me and help make sense of things. Everything was in one place which was convenient” (ID5)
		Free writing	“I preferred paper once I got going. I liked to see my maps and patterns as I wrote. I referred back to them. I found the journaling therapeutic, physically writing worked for me” (ID6) “I would have liked more free text journaling. I found that really helpful on paper” (ID8)
		Restrictive	“I found the targets were too simple for my case. My issues were more complex and the app couldn’t be tailored as much as I needed” (ID8)
		Ambiguity	“When it came to recognitions I was confused – not seen – fully recognized. What if it didn’t apply that day?” (ID7) “The scales need numbering or more detail. What is the middle of a scale? It’s too ambiguous” (ID5)
		Creative additions and personalization	“It would be good to be able to zoom in on areas of the map. Have it be more interactive with therapists notes/audio, reminding me of the focus and available coping or exits” (ID4) “I’d like the exits to link to a storage of best coping strategies. So they are just there right away for you to draw on” (ID4) “I would have liked to change the colors of the app to make them more welcoming” (ID2) “If you could create a character in the app, to guided you, engage with, that gets to know you, that would be good” (ID9)

^a^Cognitive analytic therapy.

A benefit of the technology identified by therapists was the app was easy and unobtrusive for patients to use while prompting them to engage in relational awareness tasks. The therapists made a range of design recommendations about future iterations of the app including ability to add more diagrammatic reformulations, ability to add more than 3 target problems, modification of the recognition scale to also include “not occurred” to avoid ambiguity, nudges/incentives for change, rewards to reinforce relational change, additional journaling functions, addition of CORE-OM outcome scores, needing to be available offline, and also design improvements to color and style.

All 6 of the patient participants reported that they were frequent users of “apps” relating to social media, health and wellness, games, banking, and information (news, weather). All of the patients stated that they found the CAT-App easy to access and use, with no setup challenges reported. With respect to data protection and storage, 3 patients commented on the fact that they had no issues with agreeing to data sharing and storage, as this is such a common requirement for app usage. No other patient mentioned data storage or protection.

One patient reported that they would like to add some security to accessing the CAT-App on their phone, in addition to the personal identification number, such as adding date or birth or a fingerprint access (ID5). The limited functionality of the sequential diagrammatic reformulations hindered the use of the app for some patients. However, the easy, 24-hour access availability of the app did support some patients in practicing relational awareness unobtrusively on their phones and in the moment. A difference emerged between patients who preferred free-text writing and journaling and those who preferred tracking and quantifying change, with the former disengaging earlier from using the app. With respect to personalization, the ability to customize scales and tracking within the app was a feature that all patients mentioned they would find appealing. In terms of future development of the technology, the need for motivation to use the app was an issue, with reminders and messages being thought to be of potential use.

## Discussion

This project concerned a pragmatic, real-world clinical case series evaluation of a new electronic mental health app to support the delivery of an integrative form of psychotherapy. The use of apps to support integrative psychotherapies is an innovation in the field. The app was designed to enable relational awareness and change, making it distinct in terms of aims and content to the plethora of CBT-based apps available [3]. The CAT-App particularly sought to improve and innovate on extant in and out of session paper-based tools. The recognition phase of CAT was particularly well suited to electronic mental health adaptation, due to the emphasis on building relational awareness through regularly reflection on those roles and patterns maintaining contemporary distress. The content and processes of the app were therefore theoretically mapped onto the middle and final phases of CAT. This was because some electronic mental health has been criticized for not considering the manner in which the technology can be well integrated into routine care pathways [26]. However, the dropout rate in this study (30%) was higher than that reported in traditional delivery of the therapy (15%) [21]. The feasibility of using the app in clinical practice was supported by the brief (ie, 2 hour) training that therapists undertook.

Dynamic and real-time comparisons of recognition, revision, and mood ratings were not possible when using the extant paper-based recognition methods [18], and so patients received enhanced feedback to support their relational recognition efforts when using the app. The technology improved the old, paper-based approaches through being unobtrusive, containing more feedback features, capturing mood on validated scales, and storing the personal reformulations securely in one place. This would therefore be an example of technology being able to improve and innovate on previously widely used/accepted clinical methods. If the aim of recognition is to enable “a reflective and observing self” [19] that can then direct effective change, then the technology would appear a promising clinical tool in the support of this endeavor.

The aim of the CAT-App was not to replace the therapist and the therapy, but rather enhance the relational awareness work of the patient, and this aim was echoed in the interviews conducted with therapists. Therefore, therapy was still necessary alongside patient’s engagement with the app, with the two working symbiotically. The CAT-App is therefore an example of “blended digital treatment” [37] rather than “standalone” electronic mental health, as the technology was embedded into the CAT clinical care pathway [38]. The promise of blended approaches is that technology increases treatment efficiency by completing some tasks normally completed by therapists [39]. The app offered a time saving ability to therapists by having the ratings precollected and analyzed, thus circumventing the need to produce ratings in sessions. Additionally, as the app contained highly idiographic information (ie, target problems, target problem procedures, narrative reformulation, sequential diagrammatic reformulations, and goodbye letters), this would be an example of personalized medicine [40].

The question of how, when, and where electronic mental health is best used during psychological interventions is particularly relevant with regards to the generation of “digital phenotypes” that can predict impending relapse, and so effectively step in and support early intervention [41]. The promise of electronic mental health is particularly highlighted in terms of its potential to increase access to mental health services, but if the technology is purely standalone, then there is a risk that patients feel that they are being offered a “down-graded version” of face-to-face psychological work. Electronic mental health app users often abandon the technology after a brief intense period of usage, due to data inputting burden, loss of interest, and the lack of inclusive features [42]. This may have been the case here because those patients that dropped out of therapy did not use the app to any significant degree and also dropped out in the early stages of therapy.

In the context of a relational therapy such as CAT, it has been previously noted that electronic mental health users do use relational concepts when describing the technology, such as being open with and forming a bond with the technology [27]. There is a need for the “therapeutic relationship” between user and technology to be effective [43], and there have been calls for more intensive and dynamic measurement of the “therapeutic relationship” during ehealth interventions [44]. It has been argued that developers should pay far greater attention to developing and maintaining the relationship the patient has with the technology and that apps should involve an initial “relationship building” aspect [27]. The current study has highlighted that encouragement and prompts to continue with app usage would be helpful. On the other hand, promoting overly close or enmeshed relationships with technology can have potential unintended negative effects. For example, it is apparent that technology use has expanded and proliferated in most people’s everyday life [45], running the risk of interfering and intruding into relationships (“technoference”). The unintentional shadow cast by electronic mental health is that accessing the technology may actually disrupt or interfere with close relationships.

In terms of study limitations, while the size of the sample in the case series was appropriate for an early practice-based study, it still represents a study limitation in terms of being a small sample size. For example, the large effect size (>1.5) needs to be interpreted with due caution, due to the unreliability of Cohen d in small samples [46]. All the inferential statistics reported therefore need to be treated with prudence due to the small sample size, particularly with respect to the dropout rate reported. There was an acknowledged convenience selection bias in the recruitment of patients that could have been corrected through random sampling of referrals. Similarly, potential participants feeling less confident with the offer of an app may have excluded themselves from the study [47]. Other sources of selection bias were the need to own a smartphone and willingness to use the app, all the patients being white adults and the study being conducted in private psychotherapy practice. These issues of context and selection bias mean that the results may not generalize to other populations including specific ethnic groups, children or the elderly, or public health settings. There was no control group using the traditional paper-based recognition tools of CAT to compare app outcomes against. Clinical outcome measurement was limited to single outcome measure and could have been broader. The app is currently only available in English and as CAT is now practiced internationally [21], sister versions in other languages need to be developed quickly [48]. The promise of standalone and blended electronic mental health is pronounced in developing countries where funding for mental health provision may be piecemeal [49].

The study did not contain a follow-up period and therefore the degree to which the app was used beyond the end of CAT was not recorded. Capturing app usage during follow-up would be a goal in future studies, particularly as structured follow-up support is integral to the CAT clinical model [19]. When services are commissioned only to deliver acute-phase treatment, then no follow-up support is made available in the continuation phase [50], and so the potential role of technology supporting patients during follow-up appears particularly important to consider. The competence with which therapy was delivered could have been assessed using the competence in CAT measure [51]. Using the app during therapy does not change the key competencies of the CAT model [24], and this needs to be evidenced in future studies. The lack of interviewing of patients that dropped out did not enable their particular difficulties with the app to be understood.

In terms of future directions, the CAT-App needs to go through another design and content iteration assessment in order to act on the feedback received. The study was conducted in private psychotherapy practice and so evaluation in public mental health services is now indicated. Further research is needed to understand how specific and different diagnoses affect app uptake, adherence, and outcomes. For example, patients in the midst of a major depressive episode may find it difficult to use technological support due to the well-evidenced problems with attention and concentration that can interfere with people’s ability to use or interact with technology [52]. CAT principles have been applied to a 6-session guided self-help intervention for delivery at step 2 of Improving Access to Psychological Therapies services in the UK [53], and finding ways in which the app could integrate with this psychoeducational approach would be particularly useful. Similarly, the utility of the app with patients with personality disorders could be evaluated as the recognition phase of CAT is prolonged with this patient group [54]. CAT is also delivered as a one session “personal reformulation” to health professionals to help develop better professional role repertoires, and a new version of the app needs to be developed to support personal reformulations. The gamification of electronic mental health offers promise in the design of innovative delivery platforms to children and young people [55].

In conclusion, this project aimed to use a mixed methods case series design to evaluate an app designed to map onto the theoretical stages and content of a widely practiced and integrative form of psychotherapy [21]. We have learnt from this study that the recognition and revision phases of CAT can be supported using mobile technology to support patients practicing relational awareness. Such awareness has previously been illustrated to be the plinth upon which change occurs [25]. Any efforts to make relational awareness tools more accessible and less obtrusive to patients between sessions and also more time efficient for therapists within sessions are at a premium. As the app provided detailed ongoing feedback absent from the previous paper-based methods, this represents a technology-enabled advance in clinical practice. Further larger and more controlled studies are now indicated, and it may be possible to progress onto a patient preference clinical trial in which app-assisted CAT is compared against routine CAT [56]. Although the app appears to hold clinical promise, future development and associated outcome studies are also clearly indicated.
