# Correction: Evaluation of a Mobile App to Enhance Relational Awareness and Change During Cognitive Analytic Therapy: Mixed Methods Case Series

**DOI:** 10.2196/27159

**Published:** 2021-01-15

**Authors:** Stephen Kellett, Katherine Easton, Martin Cooper, Abigail Millings, Melanie Simmonds-Buckley, Glenys Parry

**Affiliations:** 1 Sheffield Health & Social Care NHS Foundation Trust University of Sheffield Sheffield United Kingdom; 2 University of Sheffield Sheffield United Kingdom; 3 Sheffield Hallam University Sheffield United Kingdom; 4 Catalyse, UK Sheffield United Kingdom

In “Evaluation of a Mobile App to Enhance Relational Awareness and Change During Cognitive Analytic Therapy: Mixed Methods Case Series” (JMIR Ment Health 2020;7(12):e19888) the authors noted one error.

The paper was inadvertently published with an incorrect list of affiliations. The original paper listed the authors and affiliations as follows:

Stephen Kellett^1^, BSc, MSc, D Clin; Katherine Easton^2^, BSc, MRes, PhD; Martin Cooper^3^, BA, MSc; Abigail Millings^2^, BSc, PhD; Melanie Simmonds-Buckley^2^, BSc, PhD; Glenys Parry^2^, BA, PhD

^1^Sheffield Health & Social Care NHS Foundation Trust, Clinical Psychology Unit, University of Sheffield, Sheffield, United Kingdom

^2^Catalyse, UK, Sheffield, United Kingdom

^3^Software Engineering, Graphics and Multimedia; Sheffield Hallam University, Sheffield, United Kingdom

The corrected affiliations are as follows:

Stephen Kellett^1^, BSc, MSc, D Clin; Katherine Easton^2^, BSc, MRes, PhD; Martin Cooper^3^, BA, MSc; Abigail Millings^2^, BSc, PhD; Melanie Simmonds-Buckley^2^, BSc, PhD; Glenys Parry^4^, BA, PhD

^1^Sheffield Health & Social Care NHS Foundation Trust, University of Sheffield, Sheffield, United Kingdom

^2^University of Sheffield, Sheffield, United Kingdom

^3^Sheffield Hallam University, Sheffield, United Kingdom

^4^Catalyse, Sheffield, United Kingdom

The correction will appear in the online version of the paper on the JMIR Publications website on January 15, 2021, together with the publication of this correction notice. Because this was made after submission to PubMed, PubMed Central, and other full-text repositories, the corrected article has also been resubmitted to those repositories.

